# Isolation and Pathogenicity of a Chinese Porcine Astrovirus Type 5 Strain HNPDS-01 and Its Influence on Cecum Microbiota in Piglets

**DOI:** 10.1155/2024/5777097

**Published:** 2024-01-30

**Authors:** Zehui Li, Xingyi Wu, Yunfei Zhang, Qianqian Li, Junlong Gao, Yixin Hu, Jin Yuan, Hui Hu, Xiaohui Jin, Zhanyong Wei

**Affiliations:** ^1^The College of Veterinary Medicine, Henan Agricultural University, Zhengzhou 450002, Henan, China; ^2^Ministry of Education Key Laboratory for Animal Pathogens and Biosafety, Zhengzhou 450002, Henan, China; ^3^Key Laboratory for Animal-Derived Food Safety of Henan Province, Zhengzhou 450002, Henan, China

## Abstract

Astroviruses have frequently been found in mammals and poultry, but only a few have been successfully isolated for extensive research. Here, we isolated a strain of porcine astrovirus type 5 (PAstV 5) on LLC-porcine kidney (LLC-PK) cells, from the intestinal contents of diarrhea piglets, namely PAstV 5-HNPDS-01. The complete genome sequence length of this strain was 6,419 nt, which has 77.2%–91.1% nucleotide homology with other PAstV 5 strains and 45.0%–50.0% nucleotide homology with other mammalian astroviruses. The recombination analysis indicated that the recombination events were occurred in ORF 2 region (4,444–5,323 nt) in PAstV 5-HNPDS-01 strain. Subsequently, the pathogenicity of PAstV 5-HNPDS-01 was evaluated in 5-day-old piglets. It showed that the PAstV 5-HNPDS-01 could cause mild diarrhea, growth retardation, minor damage to intestinal villi clinically. Meanwhile, PAstV 5-HNPDS-01 infection could affect the microbiota diversity and composition of cecum in piglet from phylum to genus level. After infected with PAstV 5, there was a significant downregulation of beneficial bacteria, including *Faecalibacterium*, *Bacteroides*, *Lactobacillus*, and *Prevotella*, while harmful bacteria such as *Subdoligranulun* showed a significant upregulation. These results provided a research basis for pathogenic mechanisms, vaccine development, and beneficial symbiotic bacteria development for PAstV 5 infection.

## 1. Introduction

Astroviruses (AstVs) belong to the family *Astroviridae*, which includes *Mammalian astrovirus* and *Avian astrovirus* [1]. Currently, AstVs contain 33 *Mamastrovirus* and 7 *Avastrovirus* species, and they were widely detected around the world within many hosts, including humans, pigs, dogs, roe deer, turkey, bats, and other host animals [2–7]. AstVs are positive-sense, single-stranded, and nonenveloped RNA viruses with a genome length of 6.4–7.3 kb [8], consisting of 5′ untranslated region (5′-UTR), open reading frame (ORF) 1a, ORF 1b, and ORF 2 (3′-UTR). ORF 1a and ORF 1b encode the nonstructural proteins 1ab (nsp1ab), which further process to serine protease and RNA-dependent RNA polymerase [8]. ORF 2 is located at the 3′ end of the genome, which encodes viral capsid protein.

Porcine astrovirus (PAstV) is a member of the mammalian astrovirus family and is mainly associated with gastroenteritis and neurological diseases in pigs. Mostly, PAstV infections can cause vomiting, diarrhea dehydration in piglets [9, 10]. Some infections can cause viremia and viral encephalitis, leading to growth retardation and other symptoms in piglets. At present, PAstV can be divided into five serotypes according to the difference in ORF 2 gene, including PAstV 1 to PAstV 5 [9, 11], and all of these genotypes were found to be prevalent in swine herds, especially common for the PAstV 1, PAstV 3, and PAstV 5 [9, 11, 12]. It has been proven that PAstV 1 mainly caused diarrhea and vomiting in piglets [9], while PAstV 3 caused nervous system diseases and infected the central nervous system [11, 13]. Currently, frequent detection of PAstVs in both healthy and ill pigs with significantly positive rates worldwidely, including South Korea (19.4%), the United States (64.0%), Hungary (85.7%), Italy (67.4%), and Australia (100.0%) [14–19]. In China, Xiao detected the different serotypes of PAstV in Hunan province with the positive rate of 46.3% and PAstV 5 was found to be the predominant genotype (24.8%), followed by PAstV 4 (16.1%) [20]; and in Sichuan province, Cai et al. [21] reported that the prevalence rate was 10.0% for PAstV 2% and 7.5% for PAstV 5. In Yunnan Province, the infection rate of PAstV 2 and PAstV 5 was 39.9% [22]. In the meanwhile, the coinfections of PAstV with other swine viruses, including porcine kobuvirus (PKoV), porcine teschovirus (PTV), classic swine fever virus (CSFV), porcine rotavirus (PoRV), and porcine coronavirus 2 (PCV 2) were also common in pig industry, causing much difficulties in clinical detection and diagnosis to the pig industry [12, 23]. Although there have been multiple evidences in the epidemiology of PAstVs, the PAstV epidemic strains that have been isolated in cell culture systems are very few.

The gastrointestinal tract, as the largest immune organ, plays a crucial role in the host's resistance to pathogen infections [24, 25]. Accumulating evidences suggest that enteric virus infections can induce gut microbiota dysbiosis, some viruses even utilize these bacteria to promote infection. Both virus infection and altered gut microbiota could further disturb the normal function of the gut barrier and lead to diarrhea [26–28]. Several studies reported that PDCoV or PEDV infections altered the bacterial diversity and microbial community in the colon of piglets. Some bacteria like *Mitsuokella* showed a strong correlation with proinflammatory cytokines TNF-*α*, IL-6, and IL-8 expression [26, 27]. The bacterial microbiome in the intestines of COVID-19 patients has also changed significantly, which is characterized by the reduction of beneficial symbiotic bacteria in the intestines and the enrichment of conditional pathogenic bacteria. After it was cured, the abundance of gut microbiota did not return to its original level [29]. However, there is no relevant report on the impact of PAstV 5 on piglets' intestine microbiota and little is known about the role of the gut microbiota during PAstV 5 infection.

In this study, a PAstV 5 Henan strain, HNPDS-01, was isolated and its biological characteristics were determined. The pathogenicity of PAstV 5 HNPDS-01 was evaluated in 5-day-old piglets. These results enhanced the understanding of the biological characteristics of PAstV 5. Meanwhile, 16S rRNA microbial sequencing of thececum contents from piglets infected with PAstV 5 was performed to analyze the characteristics of cecal microbiota, which provided a research basis for the screening of beneficial symbiotic bacteria.

## 2. Materials and Methods

### 2.1. Cells

The LLC-PK cells, swine testis (ST) cells, and IPEC-J2 cells were purchased from Institute of China Veterinary Medicine Inspection. The LLC-PK cells were used to isolate PAstV 5. The growth medium for LLC-PK cells were minimum essential medium (MEM, Gibco, USA) supplemented with 5% fetal bovine serum (FBS, Gibco, USA), 1% penicillin–streptomycin solution (anti–anti, Gibco, USA), 1% nonessential amino acids (NEAA, Gibco, USA), and 1% 4-(2-hydroxyerhyl) piperazine-1-erhane sulfonic acids (HEPES, Gibco, USA). The LLC-PK cells' maintenance medium is MEM containing 5 *μ*g/mL trypsin. The ST cells were cultured in Dulbecco's modified Eagle medium (DMEM, Gibco, USA), supplemented with 8% FBS (VivaCell, China) and 1% anti–anti. The ST cells' maintenance medium was DMEM containing 1% pancreatin (Gibco, USA). The IPEC-J2 cells were cultured in Dulbecco's modified Eagle medium nutrient mixture F-12 (DMEM/F-12, Gibco, USA) and supplemented with 5% FBS (Gibco, USA) and 1% anti–anti. The maintenance medium for IPEC-J2 cells is DMEM/F-12 containing 2.5 *μ*g/mL trypsin.

### 2.2. Virus Isolation and Propagation

The intestinal contents from diarrhoeal pigs were collected from a commercial pig farm in Henan, China. The sample was diluted five times with MEM, containing 1% anti–anti and then centrifuged at 3,000 rpm at 4°C for 15 min. The supernatant was collected and filtered with 0.22 *µ*m filters. The filtrate was used as the inoculum for virus isolation on LLC-PK cells.

LLC-PK cells were seeded in six-well cell culture plates. When the cell monolayers reached about 90% confluence after about 24 hr of growth, cells were washed twice with phosphate-buffered saline (PBS). The above filtrate was added to the cell culture and incubated in a 5% CO_2_ incubator at 37°C for 1 hr, then washed twice with PBS. The MEM containing 1% anti–anti, HEPES, and NEAA 5 *μ*g/mL trypsin was added into the cell culture. The culture was further incubated at 37°C in 5% CO_2_ and monitored for cytopathic effect (CPE). When the CPEs reached over 80%, the cell culture plate was frozen and thawed twice at −80°C. The virus was collected and further passaged in LLC-PK cells.

### 2.3. Virus Plaque Purification

The virus was plaque-purified on LLC-PK cells according to the reported method [30]. LLC-PK cells were seeded in a six-well plate and grew to 100% around 24 hr. After washing twice with PBS, the virus was added to each well. After adsorbed for 1 hr in 5% CO_2_ incubator at 37°C, the plates were washed twice with PBS. Then 2 mL of agarose-MEM, which contained 1.5% (w/v) seaPlaque agarose (Lonza, Rockland), 1% anti–anti 5 *μ*g/mL trypsin, were added to covered the underlying cells. After being cultured for about 24 hr, the plaque was stained with 0.01% neutral red for 20 min. The typical plaques were selected and dissolved in 0.5 mL MEM and stored at −80°C

### 2.4. RNA Extraction, Deep Small RNA Sequence Assembly

Viral RNA was extracted from intestinal contents and tissues, cell culture samples by the TRIzol method (Vazyme, China). The total RNA, which was extracted from the plaque-purified virus, was subjected to next-generation sequencing (NGS) on the Illumina platform (Tanpu Biotechnology Co., Ltd. China). After quality control (QC) of the obtained sequencing data, the clarified total RNA was used to complete the whole genome sequence and the Illumina PE library was constructed. The reads obtained from the above QC and decontamination sequence are assembled by de novo. SPAdes and MEGAHIT software assembled the second-generation data. The contigs obtained from the above assembly were compared with the virus NT database by BLAST (V2.10.0+) to determine the candidate reference sequence with the closest evolutionary relationship.

### 2.5. Transmission Electron Microscopy (TEM)

The virus passage 14 (P14) was centrifuged at 8,000 rpm for 30 min at 4°C. The supernatant was filtered with 0.45 and 0.22 *μ*m filters successively to remove cell debris. The filtrate was added with a final concentration of 5% (w/v) polyethylene glycol 8,000 (PEG 8,000) stirred with a magnetic stirrer for 2 hr at 4°C. Viral particles were pelleted by centrifugation at 140,000x *g* for 3 hr at 4°C, then resuspended in 1.5 mL PBS to wash viral particles with the same speed. The viral particles were dispersed in 50 *μ*L PBS. The 2.5% (v/v) glutaraldehyde was added to inactivate the virus for 8 min. The viral particles were adsorbed on a copper square mesh size 300. Then 2% (v/v) phosphotungstic acid staining solution for negative staining. The viral particles were observed by electron microscope (Tecnai G2 Spirit Bio, USA) after natural drying.

### 2.6. Indirect Immunofluorescence Assay (IFA)

For IFA staining, cells monolayers were washed twice with PBS and fixed with absolute ethanol for 8 hr, at 4°C. Then permeated with PBS containing 0.05% (v/v) Triton X-100 (solarbio, China) to membrane permeabilization at room temperature for 15 min. Cells were blocked with PBS containing 5% (w/v) bovine serum albumin (Sigma, USA) for 2 hr at room temperature (RT), then incubated with PAstV 5 capsid-protein-specific monoclonal antibody (6C6, prepared by our lab, 1 : 200 dilution) for 8 hr, at 4°C. After washing with PBS containing 0.05% (v/v) Tween-20 (PBST) for five times, the FITC-conjugated affinipure goat anti-mouse IgG (H + L) secondary antibody (1 : 200 dilution, Proteintech Group, Inc, China) was added and incubated around 50 min at 37°C. The plates were washed five times with PBST. The nucleus was stained with 4′, 6-diamino-2-phenylindole (DAPI) (Solarbio, China) for 10 min at RT and washed five times with PBST. Finally, cells were observed with the fluorescence inverted microscope (EVOS M5000, USA) and took photos.

### 2.7. Western Blot (WB)

For western blot, cell monolayers were washed with PBS and lysed with NP-40 with 1% (v/v) phenyl methane sulfonyl fluoride (Beyotime, China) for 20 min. Then 6x protein loading buffer (TransGen Biotech, Beijing) was added to the collected samples. Samples were separated by SDS-PAGE and transferred onto a nitrocellulose filter membrane (Cytiva, USA). The membranes were blocked with 5% (w/v) skim milk in tris-buffered saline with 0.05% (v/v) Tween-20 (TBST) for 2 hr at RT. Incubated with PAstV 5 capsid-protein-specific monoclonal antibody 6C6 (1 : 2,000 dilution) for 8 hr at 4°C. After washing three times with TBST, secondary antibody HRP–conjugated affinipure goat anti-mouse IgG (H + L; 1 : 5,000 dilution, Proteintech Group, Inc, China) was added and incubated around 1 hr, at RT. Nitrocellulose filter membrane was washed three times with PBST and incubated with ECL (Beyotime, China) to visualize protein bands.

### 2.8. Tissue Culture Infective Dose 50 (TCID_50_) Assay

TCID_50_ assay was performed to assess viral titer. Confluent LLC-PK cell monolayers in 96-well plates were inoculated with tenfold serially diluted viruses (100 *μ*L per well) at 37°C for 1 hr. After washing twice with PBS, 200 *μ*L of LLC-PK maintenance medium was added to each well. The CPE was recorded daily. Virus titers were calculated using the Reed–Muench method and recorded as TCID50 per 100 *μ*L.

### 2.9. RT-PCR and qRT-PCR Based on the PAstV 5 ORF 1a Gene

HiScript II 1st Strand cDNA Synthesis Kit (Vazyme, China) was used to synthesize into cDNA. According to the relatively conservative region of ORF1a of PAstV 5, the specific primers (PAstV 5-207-F: 5′–CCAACTCTGATCGTGATCCT–3′ and PAstV 5-207-R: 5′–TACCACCGGTTCACATTCTCTCTT–3′) were designed for RT-PCR. The RT-PCR program was as follows: 95°C for 5 min; 35 cycles of 95°C for 20 s, 58°C for 15 s, 72°C for 15 s, 72°C for 5 min, and 4°C for storing the production. The primers for qRT-PCR were PAstV 5-117-F (5′–CTTACAGGTGTGTCCAATGCATGA–3′) and PAstV 5-117-R (5′– TACCATGCATCCCGTAGA–3′) with the program of 95°C for 1 min; 40 cycles of 95°C for 5 s, 58°C for 30 s, and 72°C for 30 s.

### 2.10. Complete Genomic Sequence and Phylogenetic Recombination Analysis

Based on the result of NGS, the specific primers (Table 1) were designed with the sequence of candidate PAstV 5 (Genbank: KP747574.1). The genes were amplified by Phanta® Max Super-Fidelity DNA Polymerase (Vazyme, China). According to the manufacturer's protocol, PCR products were purified and cloned into pMD18-T vectors (TaKaRa, China) for sequencing. The complete sequence of PAstV 5 HNPDS-01 was obtained by assembling overlapping contigs followed by trimming the primer sequence. Phylogenetic trees were constructed using the neighbor-joining method in MEGA X software (http://www.megasoftware.net/) with the bootstrap analysis of 1,000 replicates. To detect possible recombination events of PAstV 5 HNPDS-01, the RDP4 package, including RDP, BootScan, GENECONV, Maxchi and Chimera, was used to analyze the potential recombination events.

### 2.11. Animal Experiment

Six 5-day-old piglets were purchased from a commercial pig farm. The virus-specific PCRs were performed to exclude the common enteric viral pathogens, porcine sapelovirus (PSV), porcine epidemic diarrhea virus (PEDV), transmissible gastroenteritis virus (TGEV), porcine delta coronavirus (PDCoV), and porcine reproductive and respiratory syndrome virus (PRRSV). The protocols for animal experiments on live piglets were approved by the Animal Care and Use Committee of Henan Agricultural University (Zhengzhou, China) and the approval number is HNND2020031012. Six piglets were randomly divided into two groups and orally infected with PAstV 5-HNPDS-01 P14 (1 × 10^7^ TCID_50_/piglet, *n* = 3). The control group was orally inoculated with 10 mL MEM culture for each piglet (*n* = 3). All piglets were evaluated daily for clinical signs and body condition.

### 2.12. Samples Collection

Fecal samples were collected daily from each piglet with sterile cotton swabs. After 4 days postinfection (dpi), piglets in both groups were euthanized. Then, heart, liver, spleen, lung, kidney, duodenum, jejunum, ileum, cecum, colon, and rectum mesenteric lymph samples were collected for pathological examination and viral distribution detection. Fresh pathological tissues were fixed in a 4% (w/v) paraformaldehyde solution at RT. The cecal content samples were collected and stored at −80°C, which was used for microbial DNA extraction.

### 2.13. Histopathology and Immunohistochemical

For histopathology and immunohistochemical staining, after 2 days of fixation, the tissues were washed with running water for 12 hr. The tissues were attached to the glass slide by dehydration, transparency, wax immersion, and section dehydration. Then, hematoxylin–eosin (H&E) staining was used. Then, the slides were examined by conventional light microscopy. Among them, the other part needs to use sodium citrate solution for antigen repair for immunohistochemical detection. Incubated with PAstV 5 capsid-protein-specific monoclonal antibody 6C6 (1 : 200 dilution) for 8 hr at 4°C, then the immunohistochemical ultrasensitive reagent kit was used for testing to test (MXB, China). Finally, the slides were reacted with 3,3′-diaminobenzidine (DAB). After hematoxylin counterstaining, neutral gum was used to seal. Finally, the slides were observed under a light microscope.

### 2.14. Microbial DNA Extraction and 16sRNA Sequencing

The total nucleic acid of microbiota in the piglets cecal contents was extracted. The community DNA fragments were sequenced by using the Illumina platform for paired-end sequencing (Shanghai Personal Biotechnology Co., Ltd, China). The specific primers were 5′–ACTCCTACGGGAGGCAGCA–3′ and 5′–GGACTACHVGGGTWTCTAAT–3′. The software package DADA 2 method [31] was used for primer removal, quality filtering, noise removal, and splicing chimerism removal and then compared with the Release 13.8 database [32]. The results were scored and judged for taxonomic annotation. Through the statistics of the characteristic table after the removal of singleton, the visualization of the composition and distribution of each sample at the six classification levels of phylum, class, order, family, genus, and species were realized.

### 2.15. Bioinformatics Analysis

The ASV/OTU abundance chart was used for community analysis. Alpha diversity (Shannon, Simpson) was used to evaluate the microbiota community. Beta diversity, based on the Bray–Curtis distance, reflected the diversity of species composition among different communities through principal coordinate analysis (PCoA). The species composition of each sample at the six classification levels of phylum, class, order, family, genus, and species was realized by a stacked bar chart (QIIME2). The LDA effect size (LEfSe) indicated the microbiota community as the significantly enriched species in each group. The minimal threshold of linear discriminant LEfSe was set at 4.

### 2.16. Statistical Analysis

All the above statistical analysis were performed with a one-way analysis of variance (ANOVA) or evaluated with a multiple *t* test. Compared with the corresponding control, the difference was significantly relative when  ^*∗*^*P*  < 0.05 and  ^*∗∗*^*P*  < 0.01.

## 3. Results

### 3.1. Isolation, Identification, and Purification of PAstV 5-HNPDS-01 Strain

**Figure 1:**
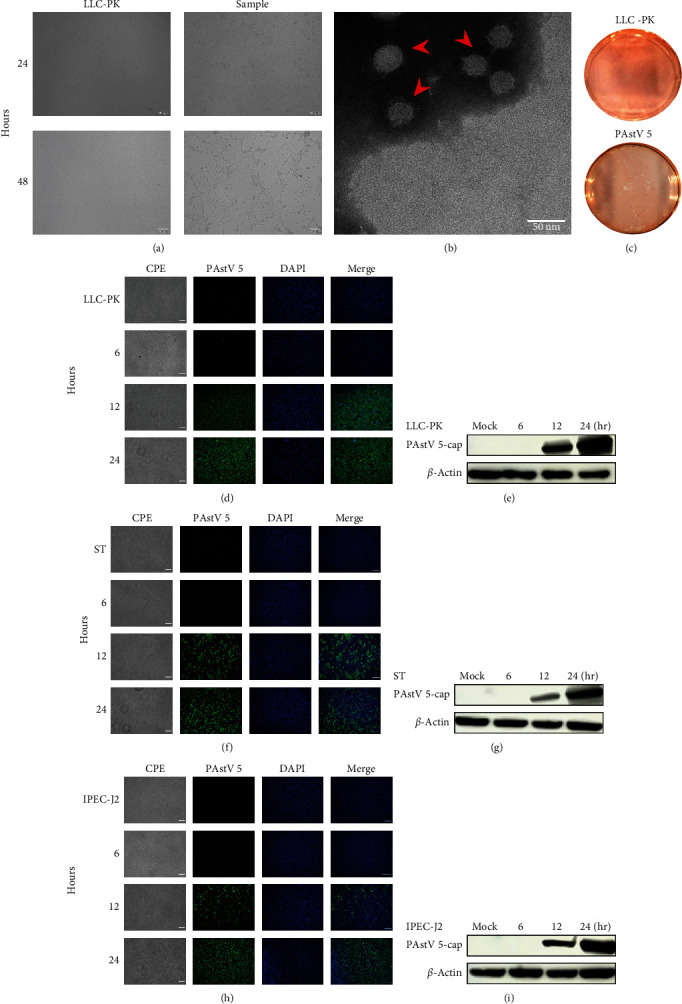
Isolation and identification of PAstV 5-HNPDS-01 strain. (a) The CPE of 544LLC-PK infected with PAstV 5-HNPDS-01 at 24, and 48 hpi, the scale bar is 100 *μ*m. (b) TEM of PAstV 5-HNPDS-01 particles, sample was negatively stained with 2% (v/v) phosphotungstic acid, the scale bar is 50 nm. (c) Plaque-purified PAstV 5-HNPDS-01547 strain. (d–i) LLC-PK cells, ST cells, and IPEC-J2 cells were infected with PAstV 5-HNPDS-01 at an MOI of 0.02 and monitored for CPE. Collecting the samples at 6, 12, and 24 hpi was performed to IF and WB (PAstV 5 capside-protein antibody prepared in our lab). The scale bar is 200 *μ*m.

LLC-PK cell monolayers were inoculated with the filtered diarrhea samples collected from the diarrheic piglets. After 24 and 48 hr postinfection (hpi), the cells showed a significant CPE, which was characterized by expansion, fragmentation, small particles detachment of the LLC-PK cells (Figure 1(a)). Thus, viral RNA was extracted from cell culture samples and the common swine enteric viruses (PEDV, TGEV, PSV, PDCoV, and porcine rotavirus) were tested by viral-specific RT-PCRs. But the CPE-positive isolate was negative for all these viruses. So, the isolated virus RNA was further sequenced by NGS on the Illumina platform. The result indicated that this isolate was PAstV 5, and no other viruses were included.

Furthermore, the viral particles in the supernatant culture were purified and concentrated. The viral particle morphology was observed by TEM. It showed that the viral particles were about 30 nm in diameter, which was consistent with the size of most astroviruses (Figure 1 (b)). The isolated virus was plaque purified on LLC-PK cells (Figure 1(c)), and the positive plaque clone was selected for further serially passage on LLC-PK cells. To further identify the replication of PAstV 5 on the LLC-PK cells, LLC-PK cells were infected with PAstV 5-HNPDS-01 at an MOI of 0.02. IFA and WB were conducted with PAstV 5 capsid-protein-specific monoclonal antibody 6C6. The infectious cells showed large numbers of IF-stained cells at 12 and 24 hpi (Figure 1(d)). PAstV 5 replication was increased dramatically at 12 and 24 hpi (Figure 1(e))

Meanwhile, the same experiment was conducted on ST and IPEC-J2 cells. The results showed that PAstV 5 was more susceptible to LLC-PK cells than that on the ST and IPEC-J2 cells at the same infective dose (Figure 1(f)–1(i)). The above results identified that a PAstV 5 strain was successfully isolated in LLC-PK cells. It was named PAstV 5-HNPDS-01 strain.

### 3.2. Replication Kinetics of PAstV 5 Strain HNPDS-01 in LLC-PK Cells

**Figure 2:**
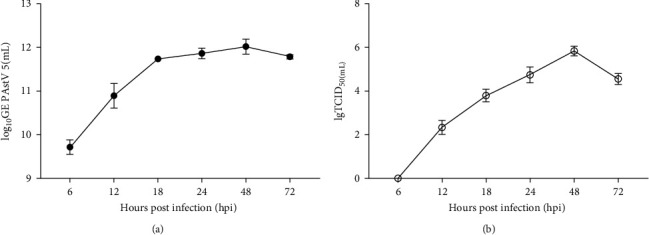
Replication kinetics of PAstV 5-HNPDS-01 in LLC-PK cells. (a) LLC-PK cells were infected with PAstV 5-HNPDS-01 at an MOI of 0.02. Samples were collected at 6, 12, 18, 24, 48, and 72 hpi. The genomic copies were detected by qRT-PCR. (b) Virus titer in LLC-PK cells was titrated with TCID_50_.

To further investigate the biological characterization of PAstV 5-HNPDS-01 strain on its sensitive cells LLC-PK, the plaque-purified PAstV 5-HNPDS-01 P14 was incubated into LLC-PK cells. Cells and supernatants were harvested together at 6, 12, 18, 24, 48, and 72 hpi. Viral gene copies and titers were detected by qRT-PCR and TCID_50_. The results showed that the gene copies of the PAstV 5-HNPDS-01 strain in LLC-PK peaked at 48 hpi (11.44 lg GE/mL; Figure 2 (a)). Its viral titer peaked at 48 hpi (5.9 lg TCID_50_/mL), then began to decline at 60 hpi (4.7 lg TCID_50_/mL; Figure 2 (b)).

### 3.3. Complete Genome Sequence and Phylogenetic Analysis of PAstV 5-HNPDS-01

**Figure 3:**
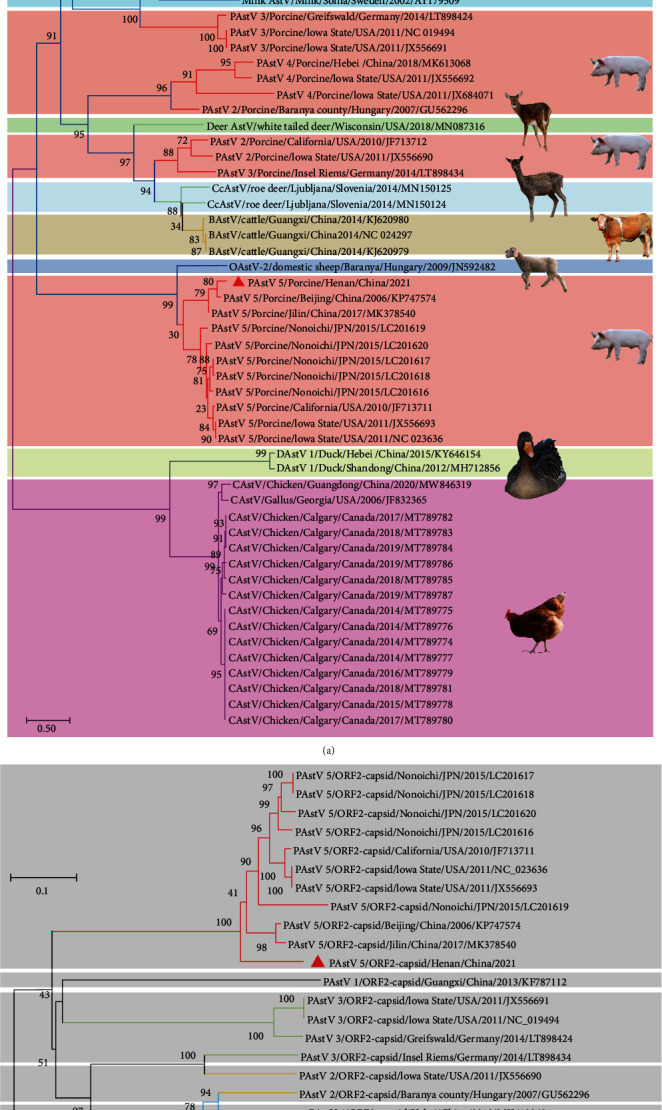
Complete genome sequence and phylogenetic analysis of PAstV 5-HNPDS-01. (a) Complete genome sequence of PAstV 5-HNPDS-01 was amplified by RT-PCR with full-sequence amplification primers (Table 1). (b) The whole genome sequence was assembled by seqMan the phylogenetic tree was subsequently constructed from the aligned sequences using the neighbor-joining method (bootstrap was 1,000 replicates) of MEGA X software. Red triangles indicated the PAstV 5-HNPDS-01. Phylogenetic tree for the ORF 2 gene was constructed with above method, of which red triangles indicate the ORF 2 of PAstV 5-HNPDS-01.

The complete genome of the PAstV 5-HNPDS-01 P10 was amplified by RT-PCR and sequenced twice (data was not show). The complete genome sequence was assembled and deposited in GenBank database (OQ781001). The full-length genome was 6,419 nt, including 5′′UTR–ORF1a—ORF 1b—ORF 2–3′UTR—poly A. Subsequently, astrovirus sequences were downloaded from the GenBank database, including human, porcine, cat, white-tailed deer, roe deer, cattle, domestic sheep, duck, and chicken, among 57 different sequences of different species. The phylogenetic tree, based on complete genome sequences, was constructed by the neighbor-joining method in MEGA X (Figure 3(a)). The results showed that the nucleotide homology between PAstV 5-HNPDS-01 with other mammalian astroviruses was 45.0%–50.0%, and with 77.2%–91.1% of the nucleotide homology with other PAstV 5 strains. All the PAstV strains were divided into five groups in the phylogenetic tree, representing the five distinct genotypes from PAstV 1 to PAstV 5 (Figure 3(a)). Furthermore, analysis of the PAstV 5 strains indicated that all the strains from Japan and U.S. clustered into a subclade, while PAstV 5 strains from China were clustered in a separately subclade. The homology analysis of ORF 2 gene of PAstVs was also found to be in general agreement with the abovementioned results, the PAstV 5-HNPDS-01 ORF2 gene clustered with the PAstV 5 group (Figure 3(b)).

### 3.4. Recombination Analysis of PAstV 5-HNPDS-01 Strain

**Figure 4:**
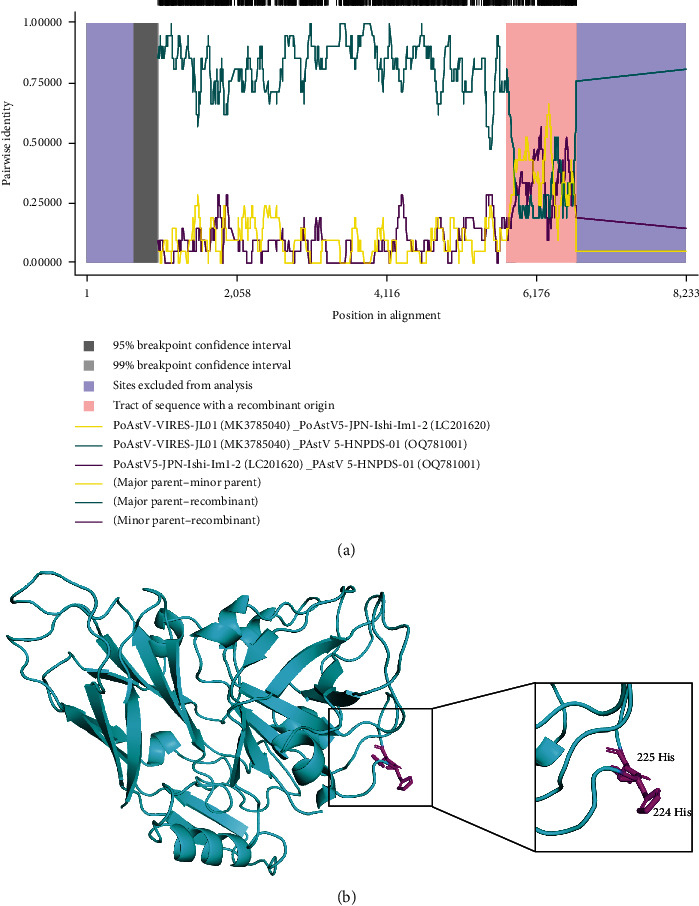
Recombination analysis of PAstV 5-HNPDS-01. (a) Analysis of recombination events in this strain by RDP4, supported by five recombination detection programs (Av. P-Val of 1.919 × 10^−04^ for RDP, GENECONVDE, BootScan Av. P-Val of N.A., Av. P-Val of MasChi was 8.152 × 10^−17^, Av. P-Val of Chimera was 3.862 × 10^−9^). (b) Homology modeling of possible recombination by swiss-model (Swiss-model). The results were visualized with Pymol.

To analyze the association between PAstV 5-HNPDS-01 strain and other PAstV strains, the recombination analysis was performed by RDP4. First, the 57 strain genome sequences, including PAstV 5-HNPDS-01 strain sequence, were compared with the RDP4. One recombination event was predicted in the PAstV 5-HNPDS-01 at 4,344–5,732 nt, when the PoAstV-VIRES-JL01 (MK378540) was used as major parent strain and the PAstV 5-JPN-Ishi-Im1-2 (LC201620) was served as the minor parent of the recombinant strain (Figure 4(a)). The sequences in the suspicious recombination region (4,344–5,732 nt) were further analyzed. The amino acid homology of PoAstV-VIRES-JL01 (MK378540) and PAstV 5-JPN-Ishi-Im1-2 (LC201620) in this region is 84.9% and 83.2%, respectively; and two histidine were inserted at the 244 and 245 amino acid position (marked as magenta sticks model) in PAstV 5-HNPDS-01 strain (Figure 4(b), Table 2).

### 3.5. Clinical Manifestations and Necropsy of Piglets Challenged with PAstV 5-HNPDS-01

**Figure 5:**
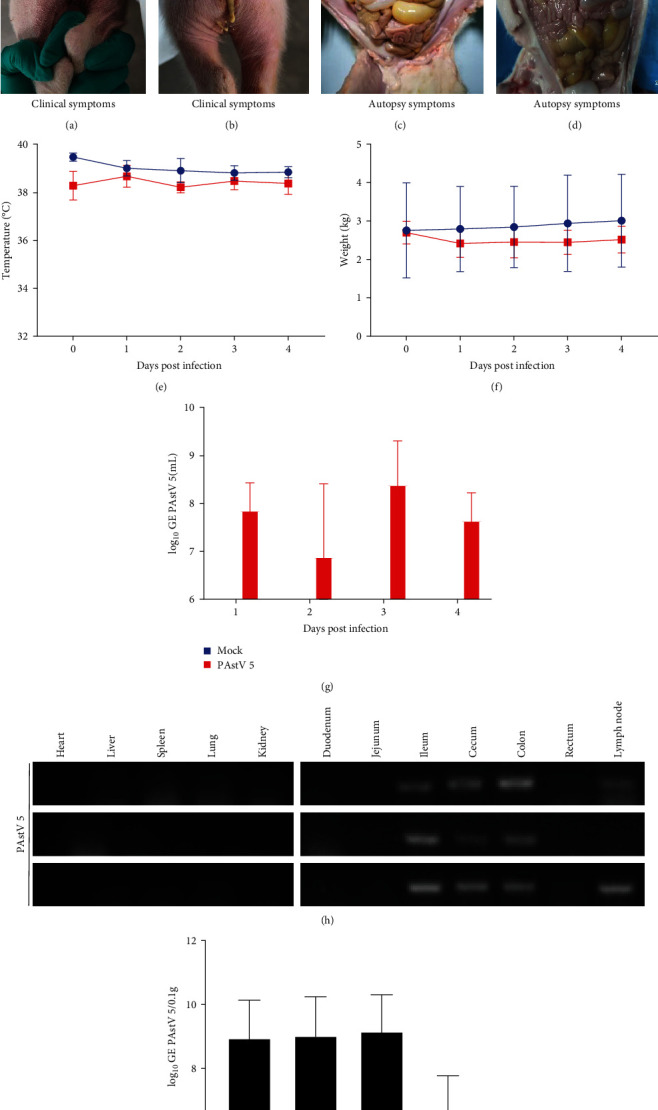
Clinical assessment of piglets infected with PAstV 5-HNPDS-01 strain and viral distributions in tissues. (a, c) 5-day-old piglets challenged with MEM medium. (b, d) 5-day-old piglets challenged with PAstV 5-HNPDS-01 strain. (e) Daily body temperature changes in piglets. (f) Daily body weight changes in piglets. (g, i) Daily fecal detoxification in piglets; (h) RT-PCR and qRT-PCR to detect virus distribution in individual tissues of piglets infected with PAstV 5-HNPDS-01. Virus copies (lg GE/0.1 g) were the average genomic copies of three piglets.

The pathogenicity of PAstV 5-HNPDS-01 was evaluated in 5-day-old piglets. The PAstV 5 infection group showed slight and transient diarrhea at 2 dpi (Figures 5(a)and 5(b)). The body temperature and weight of the two experimental groups did not show significant changes (Figures 5(e) and 5(f)). PAstV 5 RNA was detected in the fecal swabs by qRT-PCR from 1 to 4 dpi. Peak viral RNA shedding was observed at 3 dpi (Figure 5(g)). The control pigs showed no clinical signs during the study period.

The PAstV 5 infected piglets showed symptoms of yellow fluid accumulation in the intestinal tract, thinning of the intestinal wall, and slight flatulence (Figures 5(c) and 5(d)). To explore the PAstV 5 distribution in the organs of the infected piglets, the viral load in each tissue was detected by RT-PCR and qRT-PCR. The PAstV 5 nucleic acid was detected in the ileum, cecum, and colon lymph nodes of the infected group (Figures 5(h) and 5(i)). All the corresponding tissues from the control group were negative for PAstV 5.

### 3.6. Histopathology and Immunohistochemistry on the tTissue of the Piglets Inoculated with PAstV 5-HNPDS-01 Strain

**Figure 6:**
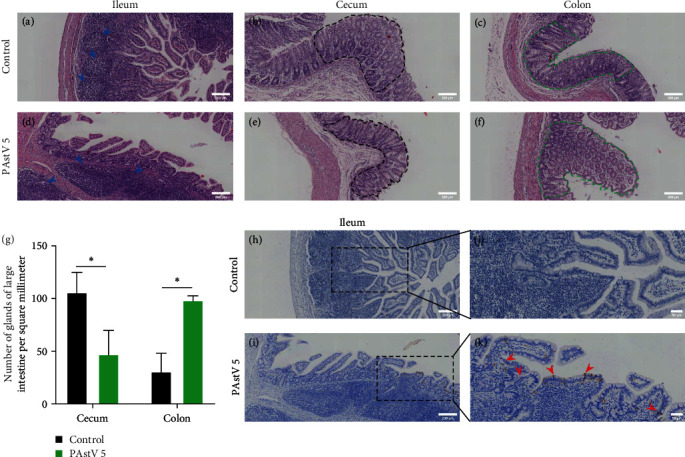
Histopathological and immunohistochemical analysis of PAstV 5 -HNPDS-01 challenged piglets. (a–f) Piglets intestinal tissues (ileum, cecum, and colon) with typical histological lesions observed by hematoxylin–eosin (H&E) staining, scale bar is 200 *μ*m. (g) Number of glands in large intestine per/square millimeter. (h–k) Piglets intestinal tissues (ileum) tested PAstV 5 specific monoclonal antibody, arrows indicate detection of PAstV 5 antigen in tissues, the scale bar is 200 *μ*m in (h) and (i), the scale bar is 200 *μ*m in (j) and (k).

Fixed intestinal tissues were detected by histopathology and immunohistochemistry. The PAstV 5 infection groups showed intestinal villi shedding and slighting atrophy (Figure 6(a)–6(f)). Compared to the control group, the PAstV 5 infection group had enlarged and fewer lymph follicles in the ileum, and the outline of lymphatic follicles was unclear (marked blue arrow, Figures 6(a) and 6(d)). In the cecum, the number of glands in the PAstV 5 infection group was significantly reduced when compared to the control group (*P*  < 0.05, marked black dotted boundary, Figures 6(b), 6(e), and 6(g)). In the colon, the number of glands in the PAstV 5 infection group significantly increased compared to the control group (*P*  < 0.05, marked green dotted boundary, Figures 6(c), 6(f), and 6(g)) in contrast to the cecum. No lesions were found in the intestines of the control piglets. PAstV 5 antigens were detected in the epithelial cells of the ileum (marked red arrow, Figures 6(i), and 6(k)), while the intestine of uninfected piglets was negative (Figures 6(h) and 6(j)).

### 3.7. The Alteration of the Microbiota Diversity in the Cecum of Piglets between the Control and PAstV 5-HNPDS-01 Infection Groups

**Figure 7:**
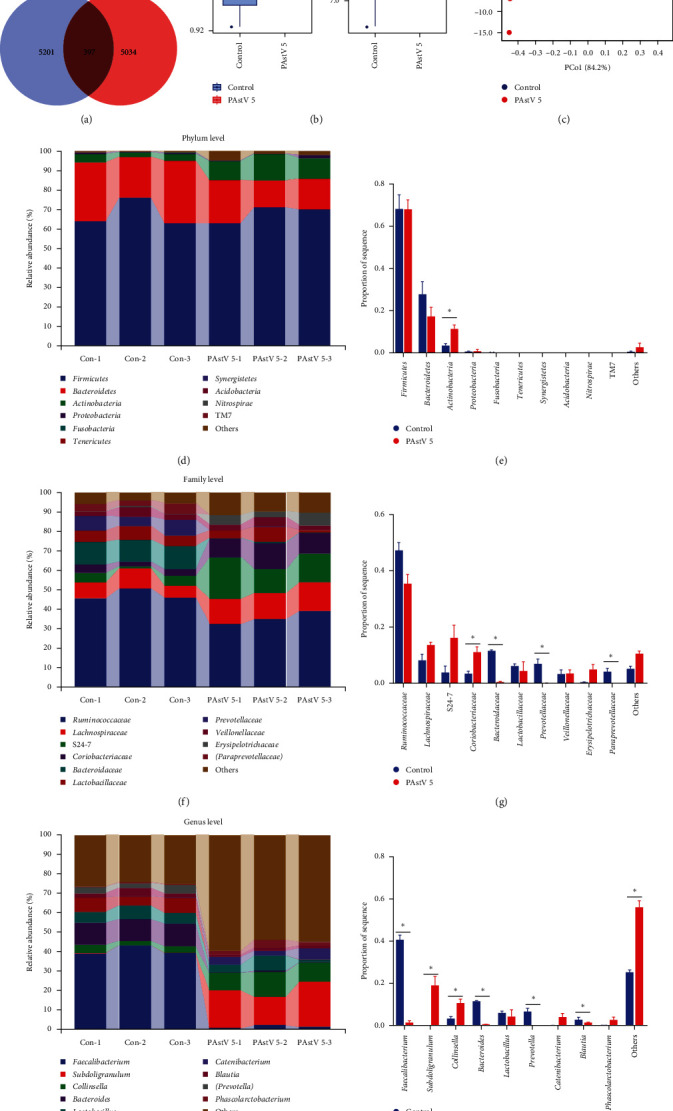
Alteration of the microbiota diversity and composition in the cecum of piglets. (a) Venn diagram of shared OTUs based on the sequences with in each group of piglets (*n* = 3). (b) The alpha diversity indexes of the cecum microbiota in piglets (*n* = 3). (c) Diversity of cecum microorganisms between control and PAstV 5 infection groups by PCoA. (d–i) Diversity of cecum microorganisms between control and PAstV 5 infection groups at the phylum, family, and genus levels. The distribution of cecum microorganism at phylum, family, and genus levels, and the distributional trends of cecum species composition differences and species abundance were analyzed by QIIME2. The statistical analysis was performed with a multiple *t* test. Compared with the corresponding control, the difference was significantly relative when  ^*∗*^*P* < 0.05 and  ^*∗∗*^*P* < 0.01.

The total microbial DNA acid was extracted from cecum contents. The community DNA fragments were double-end sequenced by the Illumina platform. The cecum contents of the control and PAstV 5-HNPDS-01 infection group were annotated with ASV/OUT for domain, phylum, order, family, genus species. The Venn diagrams showed that the number of OTUs in the control and PAstV 5-HNPDS-01 infection group were 5,201 and 5,034, respectively; whereas only 397 ODUs were commonly shared (Figure 7(a)).

The control and PAstV 5-HNPDS-01 infection group showed significant differences in cecum microbiota by using *α*-diversity analysis (Simpson, *P*  < 0.05), while the Shannon showed no significant difference in these two groups (*P*  > 0.05) (Figure 7(b)); and the *β*-diversity analysis results showed there existed significant difference in the cecum microbiota of piglets between the control and PAstV 5-HNPDS-01 infection groups (*P*  < 0.05). The PCo1 and PCo2 was 6.1% and 84.2%, respectively (Figure 7(c)). These results indicated that PAstV 5-HNPDS-01 infection significantly altered the microbial diversity in cecum of piglets.

### 3.8. The Alteration of the Microbiota Composition in the Cecum of Piglets between the Control and PAstV 5-HNPDS-01 Infection Groups

The microbial community abundance at the different levels was further analyzed. There were significant differences in the relative abundance at the phylum, family, and genus levels. At the phylum level, *Firmicutes* and *Bacteroidetes* showed no significant difference between control and PAstV 5-HNPDS-01 infection group (*P*  > 0.05). Meanwhile, *Actinobacteria* in the PAstV 5-HNPDS-01 infection group significantly increased compared with the control group (*P*  < 0.05; Figure 7(d)). At the family level, *Bacteroidaceae*, *Prevotellaceae*, and *Paraprevotellaceae* showed downregulation significantly (Figure 7(e)). Compared with the control group, another promising finding was that *Coriobacteriaceae* was showed upregulated in the PAstV 5-HNPDS-01 infection group (*P*  < 0.05; Figures 7(f) and 7(g)). At the genus level, there was a significant downregulation of *Faecalibacterium*, *Bacteroides*, *Prevotella*, and *Blautia* in the PAstV 5-HNPDS-01 infection group (*P*  < 0.05), when compared to the control group. Meanwhile, *Subdoligranulum* and *Collinsella* showed significant upregulation (*P*  < 0.05) in the PAstV 5-HNPDS-01 infection group significantly increased compared with control group (*P*  < 0.05 Figures 7(h) and 7(i)).

### 3.9. Differences of Special Taxa between the Control and PAstV 5-HNPDS-01 Infection Groups

**Figure 8:**
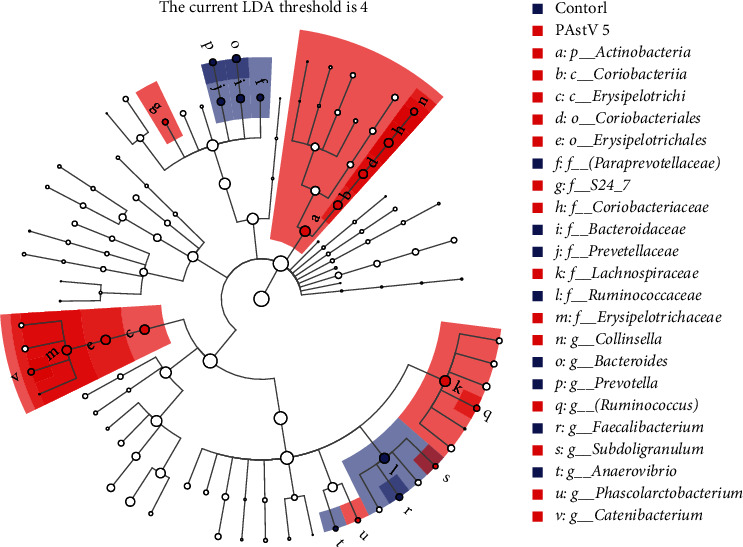
LEfSe analysis of the difference in species between control and PAstV 5 infection groups of their cecum microbe biomarker stable, with one-against-all (less strict) comparison strategy Wilcoxon test, in which the LDA threshold was set to 4.

The marker species of cecum microbial between the control and PAstV 5-HNPDS-01 infection groups were analyzed by the LEfSe. Eight potential microbial biomarkers were identified in the control group, including *Faecalibacterium*, *Ruminococcaceae*, *Bacteroidaceae*, *Bacteroides*, *Prevotellaceae*, *Prevotella*, *Paraprevotellaceae*, *Prevotella*, and *Anaerovibrio*. Meanwhile, 14 potential microbial biomarkers were identified in the PAstV 5-HNPDS-01 infection group's cecum contents, including *Subdoligranulun*, *S24-7*, *Actinobacteria*, *Coriobacteriia*, *Coriobacteriaceae*, *Collinsella*, *Coriobacteriales*, *Erysipelotrichales*, *Lachnospiraceae*, *Erysipelotrichaceae*, *Catenibacterium*, *Phascolarctobacterium*, and *Runinococcurs* (Figure 8). These results showed that PAstV 5-HNPDS-01 infection altered the special taxa of the cecum in piglets.

## 4. Discussion

Porcine enteroviruses could lead to economic losses in pig breeding industry. Viruses that do not cause serious clinical symptoms in pigs may have an impact on public health [17, 33]. Previous research showed that PAstV infections can cause vomiting, diarrhea dehydration in piglets [10, 34]. In this study, we isolated the PAstV 5-HNPDS-01 in LLC-PK from a clinical diarrheic sample and analyzed for its biological characteristics. The pathogenicity of the isolated strain on 5-day-old piglets showed that this strain caused mild diarrhea, growth retardation, and minor damage to intestinal villi clinically. Meanwhile, PAstV 5-HNPDS-01 infection significantly altered the microbial diversity and community abundance in cecum of piglets.

Many astroviruses did not produce regular CPE in cell culture, which caused many difficulties in virus isolation. Among these, Lee and Kurtz [35] first found that human astrovirus could multiply replication on human embryonic kidney (HEK) cells under the condition of adding exogenous trypsin; and a PAstV strain was isolated from pigs with acute gastroenteritis and showed obvious CPE, which was characterized by enlarged cells and fine particles in the cytoplasm [36]. The majority of previous isolates of PAstV have come from samples of pigs with different clinical signs. In this study, we successfully isolated a PAstV 5 strain from the clinical sample of diarrhea pigs by adding exogenous trypsin. It had a significant CPE, which was characterized by expansion, fragmentation, and small particles detachment on the LLC-PK cells. The LLC-PK adapted-cultured strain (PAstV 5-HNPDS-01) titer was 5.5 lg TCID_50_/mL was more sensitive to LLC-PK than ST or IPEC-J2 cells.

PAstV is mainly related to gastroenteritis and nervous system disease in piglets [9, 10]. Due to the characteristics of PAstV 5 genome that is easy to mutate, the risk of cross-species transmission of PAstV 5 and the occurrence of more pathogenic mutations and recombination cannot be ignored [15, 16]. Compared with mammalian astrovirus, avian astrovirus has stronger pathogenicity, which mainly causes viral hepatitis and nephritis in birds [37, 38]. Because of the high mutation rate of genomics, there is also the possibility of transmission from birds to mammals [15, 16]. Recombination events and mutations in RNA viruses are one of the main factors determining the molecular evolution of RNA viruses. The results showed that the nucleotide homology between this strain and other PAstV 5 strains was 77.2%–91.2% in ORF 2 gene.

Previous research showed that symbiotic microbiota can prevent pathogens from invading create a microenvironment that is adverse to intestinal pathogens [39, 40]. After PAstV 5-HNPDS-01 infection, the microbial composition of cecum in piglets was altered significantly from phylum to genus level. These results showed that the cecum microbial community and composition were significantly different between the control group and the PAstV 5-HNPDS-01 infection group. *Bacteroidota*, *Firmicutes*, and *Actinomycetes* are all dominant bacteria in the gut microbiota of mammalian [41]. However, compared with the control group, there was a significant increase in *Actinomycetes* in the PAstV 5-HNPDS-01 infection group. At the family level, *Coriobacteriaceae* showed a significant upward trend in the PAstV 5-HNPDS-01 infected group, while *Bacteroidaceae*, *Prevoteaceae*, and *Paraprevoteaceae* showed a significant downward trend. *Actinomycetes and Coriobacteriaceae* had the effect of reducing the oxidation reaction in animals with intestinal injury, which was negatively correlated with inducible nitric oxide synthase (iNOS) and short-chain fatty acid (SCFA) [28]; that can conducive to the recovery of the intestinal barrier [28]. At the genus level, compared with the control group, *Faecalibacterium*, *Bacteroides*, *Lactobacillus*, and *Prevotella* were significantly downregulated in the PAstV 5 infection group, while *Subdoligranulun* and *Collinsella* were significantly upregulated. *Faecalibacterium* is one of the most important bacteria in the microbiota, accounting for 5%–15% of the total number of bacteria detected in healthy animals [42]. It is one of the important producers of butyric acid. It has anti-inflammatory effects, maintains bacterial enzyme activity protects the digestive system from intestinal pathogens [42]. Besides, *Collinsella* produces ursodeoxycholic acid in the intestinal tract, which can prevent the binding of COVID-19 to infection receptors and inhibit inflammation, cellular decay oxidation. Its increase may inhibit pathogen invasion to some extent [43]. Others, including *Bacteroides*, *Prevotell*, and *Blautia* are also beneficial bacteria, which jointly build the intestinal microbial barrier. But the PAstV 5-HNPDS-01 infected group showed significant downregulation. The results of the experiment found clear support for the PAstV 5-HNPDS-01 destroyed the intestinal flora balance of piglets, and it had the function of self-protection and repair. Our results indicated that *Subdoligranulun* was significantly upregulated in the PAstV 5-HNPDS-01 infected group. A previous study has reported that this strain may cause rheumatoid arthritis (RA) in high-risk individuals with clonal IgA and IgG auto-antibodies [44]. From these results, it is clear that PAstV 5-HNPDS-01 was obvious to change the intestinal barrier and gut microbiota of piglets.

In summary, a strain of PAstV 5 named HNPDS-01 was isolated successfully and its biological characteristics was analyzed. Recombination analysis showed that the genome may be recombined at 4440–5737 nt in ORF 2 region. The pathological autopsy revealed yellow exudate, abdominal gas, and high viral loads in the intestine. In addition, there were significantly different in the cecum microbiota from phylum to genus levels in the PAstV 5-HNPDS-01 infected and the control groups. These results may help to understand the pathogenicity of PAstV 5, and the intestinal microbiota associated with PAstV 5 infection. Meanwhile, the influence on cecum microbiota in piglets may provide a research basis for beneficial symbiotic bacteria.

## Figures and Tables

**Table 1:** The primer information for the complete genome of PAstV 5-HNPDS-01.

Primers'	Sequences' (5′−3′)	Products' (bp)
PAstV 5-F1	TTTAAGGCTTGCGTGGTGGAGGGTTT	395
PAstV 5-R1	TGCGTGGTGGAGATCTTTCTCTTTCGT	

PAstV 5-F2	TATGATAACGCGTACCCGTTA	2,314
PAstV 5-R2	GTGAAAATGGGCGTATACCA	

PAstV 5-F3	GACGCTTCACTGGGATCAGA	1,417
PAstV 5-R3	ATGATGTCATCAGGATCAGGAC	

PAstV 5-F4	CTGGGAGTTCTTGGACGATG	1,883
PAstV 5-R4	CACCAATTAGACTGATCTG	

PAstV 5-F5	TGATGCAGAATTGAACAACCCTGT	1,149
Oligo-dT	TTTTTTTTTTTTTTTTTT	

**Table 2:** Amino acid divergence between the sequence (4,344−5,734 nt) of PAstV 5-HNPDS-01 strain and the suspected recombinant parental sequence.

Amino acid sites	58	110	114	178	181	186	189	191	197	206	209	213	214	221	224
PAstV 5 HNPDS-01 (OQ781001)	V	G	M	R	S	K	A	E	N	S	R	L	L	Q	H
PoAstV-VIRES-JL01 (MK378540)	I	G	L	K	N	T	P	H	N	T	K	A	A	Q	—
PAstV 5-JPN-Ishi-Im1-2 (LC201620)	V	S	L	R	S	T	P	E	K	S	K	P	P	P	—

Amino acid sites	225	226	227	229	230	244	245	246	249	250	265	267	276	278	279

PAstV 5 HNPDS-01 (OQ781001)	H	L	T	S	P	I	K	V	S	E	I	V	N	V	D
PoAstV-VIRES-JL01 (MK378540)	—	V	T	S	P	V	R	V	S	G	M	A	T	I	N
PAstV 5-JPN-Ishi-Im1-2 (LC201620)	—	S	A	T	T	I	K	T	A	E	M	V	T	V	N

Amino acid sites	280	281	282	285	288	290	298	303	305	306	307	311	313	315	321

PAstV 5 HNPDS-01 (OQ781001)	A	A	S	G	I	A	N	T	L	S	A	E	K	D	M
PoAstV-VIRES-JL01 (MK378540)	A	S	T	A	V	T	N	S	I	A	P	D	N	D	I
PAstV 5-JPN-Ishi-Im1-2 (LC201620)	L	A	E	G	I	A	D	S	L	A	A	D	K	N	M

Amino acid sites	327	329	332	333	336	340	341	344	346	348	350	352	353	358	359

PAstV 5 HNPDS-01 (OQ781001)	V	L	G	T	S	V	V	S	S	A	I	A	S	T	Y
PoAstV-VIRES-JL01 (MK378540)	A	V	A	S	A	P	D	N	T	S	L	T	T	P	Y
PAstV 5-JPN-Ishi-Im1-2 (LC201620)	V	V	A	S	A	P	T	N	T	A	L	T	T	P	S

Amino acid sites	360	361	362	363	364	365	368	374	375	377	384	386	387	388	389

PAstV 5 HNPDS-01 (OQ781001)	T	G	G	I	I	E	S	N	T	T	S	N	S	E	P
PoAstV-VIRES-JL01 (MK378540)	S	R	G	L	L	H	S	S	S	T	V	S	S	E	T
PAstV 5-JPN-Ishi-Im1-2 (LC201620)	N	R	G	I	L	H	A	S	S	I	T	G	T	E	T

Amino acid sites	390	393	395	400	401	403	405	408	413	416	418	422	432	436	439

PAstV 5 HNPDS-01 (OQ781001)	L	N	L	I	S	P	I	F	V	I	D	A	N	R	T
PoAstV-VIRES-JL01 (MK378540)	Y	H	L	V	P	N	L	L	S	P	A	I	S	N	A
PAstV 5-JPN-Ishi-Im1-2 (LC201620)	S	H	Q	I	P	S	L	L	A	P	A	V	S	S	A

Amino acid sites	441	444	451	—	—	—	—	—	—	—	—	—	—	—	—

PAstV 5 HNPDS-01 (OQ781001)	V	I	D	—	—	—	—	—	—	—	—	—	—	—	—
PoAstV-VIRES-JL01 (MK378540)	A	L	E	—	—	—	—	—	—	—	—	—	—	—	—
PAstV 5-JPN-Ishi-Im1-2 (LC201620)	A	L	D	—	—	—	—	—	—	—	—	—	—	—	—

## Data Availability

Data for this research article are available upon request (weizhanyong@henau.edu.cn).
